# Virulence Mechanisms of *Mycobacterium abscessus*: Current Knowledge and Implications for Vaccine Design

**DOI:** 10.3389/fmicb.2022.842017

**Published:** 2022-03-03

**Authors:** Kia C. Ferrell, Matt D. Johansen, James A. Triccas, Claudio Counoupas

**Affiliations:** ^1^School of Medical Sciences, Faculty of Medicine and Health, The University of Sydney, Camperdown, NSW, Australia; ^2^Tuberculosis Research Program, Centenary Institute, Sydney, NSW, Australia; ^3^Centre for Inflammation, Centenary Institute, University of Technology, Sydney, NSW, Australia; ^4^Faculty of Science, School of Life Sciences, University of Technology, Sydney, NSW, Australia; ^5^Sydney Institute for Infectious Diseases and the Charles Perkins Centre, The University of Sydney, Camperdown, NSW, Australia

**Keywords:** *Mycobacterium abscessus*, cystic fibrosis, vaccines, virulence factors, reverse vaccinology

## Abstract

*Mycobacterium abscessus* is a member of the non-tuberculous mycobacteria (NTM) group, responsible for chronic infections in individuals with cystic fibrosis (CF) or those otherwise immunocompromised. While viewed traditionally as an opportunistic pathogen, increasing research into *M. abscessus* in recent years has highlighted its continued evolution into a true pathogen. This is demonstrated through an extensive collection of virulence factors (VFs) possessed by this organism which facilitate survival within the host, particularly in the harsh environment of the CF lung. These include VFs resembling those of other Mycobacteria, and non-mycobacterial VFs, both of which make a notable contribution in shaping *M. abscessus* interaction with the host. *Mycobacterium abscessus* continued acquisition of VFs is cause for concern and highlights the need for novel vaccination strategies to combat this pathogen. An effective *M. abscessus* vaccine must be suitably designed for target populations (i.e., individuals with CF) and incorporate current knowledge on immune correlates of protection against *M. abscessus* infection. Vaccination strategies must also build upon lessons learned from ongoing efforts to develop novel vaccines for other pathogens, particularly *Mycobacterium tuberculosis* (*M. tb*); decades of research into *M. tb* has provided insight into unconventional and innovative vaccine approaches that may be applied to *M. abscessus*. Continued research into *M. abscessus* pathogenesis will be critical for the future development of safe and effective vaccines and therapeutics to reduce global incidence of this emerging pathogen.

## Introduction

The *Mycobacterium abscessus* clade are an emerging group of prominent human pathogens. Comprised of the three subspecies *M. abscessus* subsp. *abscessus*, *M. abscessus* subsp. *bolletti*, and *M. abscessus* subsp. *massiliense* and previously including closely related species *Mycobacterium chelonae*, these organisms are responsible for severe skin, soft tissue, and pulmonary infections (Adekambi et al., 2017; Ryan and Byrd, 2018). The *M. abscessus* clade is particularly problematic for individuals with enhanced susceptibility to pulmonary infection, such as those with bronchiectasis or chronic obstructive pulmonary disease (COPD), prior tuberculosis (TB) infection, or individuals with cystic fibrosis (CF; Griffith et al., 2007). The incidence of NTM infection in individuals with non-CF bronchiectasis is particularly high, with an estimated 37% of all patients with this condition having an NTM infection (Mirsaeidi et al., 2013). CF patients are particularly susceptible to NTM infection, with incidence rates in one study reported to be 1,000 times higher than in the general population (Martiniano et al., 2019). CF is a recessive genetic disorder affecting the cystic fibrosis transmembrane conductance receptor (CFTR) and resulting in dysregulated chloride transport, with a wide affect across different organs including the lung. Individuals with CF have dysregulated mucus production and are particularly susceptible to bacterial infections that become chronic and difficult to eradicate (Cantin et al., 2015). *Mycobacterium abscessus* infection is extremely difficult to treat due to intrinsic, adaptive, and acquired antibiotic resistance traits; these result in poor treatment success rates as low as 30% depending on the subspecies (Nessar et al., 2012; Koh et al., 2014). Furthermore, the establishment of chronic *M. abscessus* infection is associated with a significant decline in lung function, which has a profound impact on patient quality of life (Esther et al., 2010; Kwak et al., 2019). Despite the significant impact of *M. abscessus* infection on susceptible populations, there is currently no vaccine available (approved or within clinical trials) for *M. abscessus*. Given both the difficulty in treating *M. abscessus* and the poor prognosis following the establishment of persistent and chronic infection, there is an urgent need to develop novel prophylactic interventions to reduce incidence of *M. abscessus* infections in at-risk populations.

The development of novel vaccines for pathogens, such as *M. abscessus*, can be guided by the characterization of novel virulence factors (VFs), molecular components which facilitate pathogen survival and persistence in the host. Bacterial VFs promote pathogen resistance to immune defenses, improve adherence or invasion of host cells/tissues or enhance survival through modification of the host environment (Wu et al., 2008). The study of virulence determinants can identify targets for the attenuation of pathogens, or proteins which themselves may be sufficiently immunogenic to be formulated into a vaccine (Ottenhoff and Kaufmann, 2012). For example, *M. tuberculosis* antigen 85 complex proteins are VFs that promote bacterial entry into host cells; the immunogenic nature of these proteins has led to their inclusion in numerous recombinant, subunit, and viral vectored vaccines (Babaki et al., 2017). Although some VFs are unsuitable for incorporation into vaccines—due to poor immunogenicity or unsuitable cellular location for immune exposure—mechanistic studies of these VFs can shed light onto host responses to infection and thus informs future vaccine studies.

While traditionally viewed as an opportunistic pathogen, there is now compelling evidence that the *M. abscessus* clade possesses hallmark characteristics of a true pathogen. Work on *M. abscessus* in recent years has shed light on the unique disease pathogenesis and VFs possessed by this clade. While *M. abscessus* has multiple well-characterized mycobacterial VFs, it also possesses non-mycobacterial VFs which share high homology with other notable CF pathogens (Ripoll et al., 2009). Given the developments in our understanding of *M. abscessus* virulence in recent years, the purpose of this review is to summarize our current knowledge on *M. abscessus* VFs, as well as recent research efforts to develop vaccines against this emerging pathogen. An overview of *M. abscessus* biology and pathogenesis in the context of non-tuberculous mycobacteria (NTM) can be found in Johansen, Herrmann (Johansen et al., 2020).

## Evolution of *Mycobacterium abscessus* Into a Human Pathogen

Many species of NTM are ubiquitous in both urban and natural environments. NTM are adept at survival in numerous habitats, such as soil, peats, and swamps, where the thick hydrophobic membrane of *Mycobacterium* spp. facilitates strong adherence to different surfaces and likely promotes survival in hostile environments (Falkinham, 2009). This resilience to different environmental conditions has allowed the permeation of NTM into urban settings, where species, such as *M. abscessus*, are isolated from potable water sources and plumbing systems (Thomson et al., 2013). Hospital outbreaks of NTM infections are frequently linked to contaminated water supplies (Tam et al., 2014; Guimarães et al., 2016); acquisition of infection from environmental sources, rather than from other infected individuals, is through to be the major transmission route by which *M. abscessus* gains entry to human hosts (Thomson et al., 2013). The prevalence of *M. abscessus* in nosocomial environments, combined with high-level resistance to many glutaraldehyde-based hospital-grade disinfectants (Burgess et al., 2017), has facilitated the establishment of opportunistic *M. abscessus* infections in both immunocompetent and immunocompromised individuals.

Given the incursion of *M. abscessus* into urban environments, such as plumbing systems and water sources, the occurrence of opportunistic NTM infections is unsurprising. However, the diverse array of VFs possessed by the *M. abscessus* clade points to a more complex evolution of this organism into a human pathogen. Importantly, NTM including *M. abscessus* have been isolated from free-living amoeba derived from urban water sources, suggesting a role for amoeba in enabling NTM persistence (Delafont et al., 2014). It has been proposed that early adaptations of *M. abscessus* to an intracellular amoeba lifestyle may have enhanced *M. abscessus* virulence and promoted survival in mammalian hosts (Thomas and McDonnell, 2007). Preculture of *M. abscessus* in *Acanthamoeba castellanii* amoeba enhances persistence *in vivo* in a murine model of infection (N’Goma et al., 2015). *Mycobacterium abscessus* also remain viable when encysted by amoeba, suggestive of both their adaptation to the intracellular lifestyle and the potential for amoeba to act as an environmental reservoir for *M. abscessus* (da Silva et al., 2018). Dubois et al. (2019) performed extensive transcriptomic analysis to track changes in gene expression induced in *M. abscessus* during intracellular growth. It was demonstrated that growth of *M. abscessus* in amoeba and macrophages induced similar patterns of differential gene expression, including an upregulation of genes to cope with intracellular stresses, such as heat shock and oxidative stress (e.g., *GroEL-ES* and *hsp*), a switch to slower growth phenotype, and utilization of fatty acids as an energy source. Following selection of *M. abscessus* genes upregulated in amoeba and subsequent expression of these genes in opportunistic pathogen *M. chelonae*, the authors showed enhanced growth of complemented *M. chelonae* in macrophages. This supports the theory that growth of *M. abscessus* in amoeba facilitates enhanced infection and survival in mammalian macrophages, and also highlights the diverse range of mechanisms *M. abscessus* uses to persist in the host (Dubois et al., 2019).

Although most NTM infections are acquired from environmental sources, phylogenetic analysis of *M. abscessus* clinical isolates suggests that CF centers may facilitate indirect person-to-person transmission of *M. abscessus* clones through fomites (Aitken et al., 2012). Whole genome sequencing of *M. abscessus* isolated in global CF centers has revealed the presence of dominant *M. abscessus* clones with diverse geographical distribution. In addition to comprising 70% of total global *M. abscessus* infections, these dominant isolates display heightened virulence in macrophages and in Severe Combined Immunodeficiency (SCID) mice (Bryant et al., 2016). This is reflective of trends observed with other well-characterized CF pathogens, such as *Pseudomonas aeruginosa*, where epidemic strains are associated with worse patient outcome (Panagea et al., 2003; Fothergill et al., 2012).

While patterns of evolution in CF pathogens have been extensively described for other species, such as *P. aeruginosa*, only recently have similar patterns been described in *M. abscessus*. Bryant et al. recently used single nucleotide polymorphism analysis of global *M. abscessus* clinical isolates to define the trajectory of *M. abscessus* evolution into a true pathogen (Bryant et al., 2021). The initial horizontal acquisition of genes from unrelated species (as described by Ripoll et al., 2009) resulted in a significant leap in *M. abscessus* genomic variation. Following this, the transition of *M. abscessus* to the preferred infection site of the lung and evolution into a pulmonary pathogen was coupled with a further increase in *M. abscessus* genomic variation. This is unsurprising given the crucible of selective pressures present within the CF lung, such as antibiotic stressors, interspecific competition, and host immune defenses all encouraging the dominance of favorable traits (Harrison, 2007; Folkesson et al., 2012). Furthermore, the physical separation of different lung lobes coupled with the heterogeneity of CF lung tissue creates numerous ecological niches that facilitate species diversification (Harrison, 2007). Bryant et al. confirmed this hypothesis with the identification of numerous, genetically distinct subclones of *M. abscessus* isolated from different areas of the lung in individual patients (Bryant et al., 2021). The authors also described the presence of *M. abscessus* clones with hypermutable phenotypes, capable of greater phenotypic variation likely due to DNA damage from the highly oxidative pulmonary environment (Ciofu et al., 2005; Martina et al., 2014). While these results identified strong evolutionary pressure on *M. abscessus* genes that promote intramacrophage survival, perhaps the most prominent finding was the apparent fitness cost of enhanced virulence of *M. abscessus* isolates. Importantly, mutants with virulence mutations display reduced transmission rates, presumably due to their impaired survival on fomites. As such, the “evolutionary potential” or continued gain of virulence of *M. abscessus* is limited provided direct patient-to-patient spread (through aerosolized droplets) does not occur (Doyle et al., 2020; Bryant et al., 2021). However, whether *M. abscessus* can spread through aerosols is still a matter that requires clarification (Bryant et al., 2013). Given the demonstrated potential of this species to acquire polymorphisms that promote *M. abscessus* survival and the possibility for continued species adaptation, there is a significant need for the continued identification of *M. abscessus* VFs.

## Mycobacteria-Specific Virulence Factors

The genus *Mycobacterium* contains approximately 200 species, many of which inhabit soil or water environments and have occasional interaction with humans (Tortoli et al., 2017; Armstrong and Parrish, 2021). However, some members of this genus, such as *M. tb* and *Mycobacterium leprae*, are extremely successful human pathogens, being the causative agents of TB and leprosy, respectively (Gupta et al., 2018). Similarly, *Mycobacterium marinum* and *Mycobacterium ulcerans* are NTM infections acquired by environmental exposure but are adept at establishing cutaneous infection in humans (Tan et al., 2020). The success of these species is tied closely with their ability to survive within the host using an extensive collection of Mycobacteria-specific VFs. As our understanding of *M. abscessus* virulence expands, there is continual discovery of virulence traits resembling other Mycobacterial species. These further contribute to the definition of *M. abscessus* as a truly pathogenic species.

### Mycobacterial Membrane Protein Large Proteins

Mycobacterial membrane protein large (MmpL) proteins are a family of VFs of the *Mycobacterium* genus, responsible for transport of lipids and siderophores to the periplasmic space (Melly and Purdy, 2019). These include complex lipids that are essential for the integrity of the mycobacterial envelope, which forms a barrier of protection from immune cells and chemotherapeutic agents (Bailo et al., 2015). Many of these lipids play prominent roles in modifying host–pathogen interactions, and as a result mutations within MmpL proteins can lead to alterations in mycobacterial virulence (Chalut, 2016).

The MmpL class of transport proteins is overrepresented in *M. abscessus*, with 31 identified MmpL proteins in the reference strain compared to 14 within *M. tb* (Deshayes et al., 2010). MmpL4 proteins have an important contribution to *M. abscessus* virulence through their transport of glycopeptidolipids (GPL) to the outer mycobacterial surface which gives colonies a glossy and smooth appearance on solid agar. Disruption of MmpL4 proteins results in the conversion from the smooth morphotype to a rough cording morphotype, the latter of which is associated with enhanced virulence due to large serpentine cord formation, triggering pro-inflammatory responses and apoptosis (Pawlik et al., 2013). Disruption of *M. abscessus* MmpL8 reduces the transport of glycosyl-diacylated-nondecyl-diols (GDND), a novel glycolipid that facilitates bacterial adhesion to macrophages and induces macrophage phagosomal rupture, and thereby reduces mycobacterial virulence (Halloum et al., 2016). MmpL proteins from mycobacterial species, such as *Mycobacterium smegmatis*, also confer resistance to some antibiotics, such as isoniazid, by acting as an efflux pump (Pasca et al., 2005; Viljoen et al., 2017). Mutations within transcriptional regulator *tetR*, which controls two MmpS-MmpL gene pairs, promote resistance to clofazimine and bedaquiline in *M. abscessus* (Richard et al., 2019), while MmpS5-MmpL5 are associated with efflux of thiacetazone derivatives (Halloum et al., 2017). Absence of either MmpL-S pair increases susceptibility of intracellular *M. abscessus* to bedaquiline, indicating the contribution of this protein to providing antibiotic resistance (Gutiérrez et al., 2019). Given the role of MmpL proteins in enhancing or attenuating virulence and conferring antimicrobial resistance, this a particularly noteworthy area of continued research for the development of *M. abscessus* vaccine candidates.

### ESX Secretion Systems

Members of the *Mycobacterium* genus possess an outer mycomembrane comprised of components that must be transported across the cell membrane to reach the extracellular space. Early secretory antigenic target (ESAT-6) secretion (ESX) systems, also known as Type VII secretion systems, are important players in facilitating transport of proteins across this outer membrane and are known to play a significant role in mycobacterial virulence, nutrient acquisition, and bacterial conjugation (Lagune et al., 2021). There are five characterized ESX secretion systems spread across the *Mycobacterium* genus, varying in complexity and in function (Gröschel et al., 2016). However, all ESX systems have a conserved set of genes that include the machinery for substrate secretion, accessory proteins, and ESX secreted proteins (Houben et al., 2014). The most extensively studied ESX system, ESX-1, facilitates *M. tb* intracellular survival *via* inhibition of phagosomal acidification and induces phagosome rupture and mycobacterial escape to the cytosol (Sturgill-Koszycki et al., 1994; van der Wel et al., 2007). The contribution of other ESX secretion systems to virulence is less well-characterized; however, the ESX-3 system of *M. tb* is known to be utilized for iron acquisition in low nutrient environments and the absence of this system attenuates growth *in vivo* (Siegrist et al., 2009; Tufariello et al., 2016). The role of the ESX-5 secretion system in slow-growing mycobacteria relates to the integrity of the mycobacterial capsule, with disruption of ESX-5 in *M. marinum* leading to inability of this pathogen to disrupt the cellular membrane (Ates et al., 2016). While attenuation of ESX-5 deficient *M. marinum* is evident in zebrafish embryos, the same mutant is hypervirulent in adult zebrafish (Weerdenburg et al., 2012). Given that zebrafish embryos solely possess innate immunity, while the adult zebrafish harbors both innate and adaptive immunity, these findings suggest that ESX-5 may play different roles in disease pathogenesis depending on whether innate or adaptive immunity is the major driver of cellular control of mycobacterial infection (Cronan and Tobin, 2014).

There are currently three characterized secretion systems of *M. abscessus*—ESX-3, ESX-4, and ESX-P. Secretion targets of ESX-3 (EsxG/H proteins) induce pro-inflammatory cytokine production when co-cultured with bone marrow-derived macrophages, and mice infected with ESX-3-deleted-*M. abscessus* display a reduced inflammatory and granulomatous response coupled with decreased bacterial survival (Kim et al., 2017). However, this work did not elucidate specific mechanisms by which ESX-3 promotes *M. abscessus* growth *in vivo*. Furthermore, this study did not include complementation of ESX-3 function in *M. abscessus*, highlighting the need for further investigation into the role of ESX-3 in *M. abscessus* virulence. In contrast, the function of ESX-4 within *M. abscessus* was recently characterized in detail (Laencina et al., 2018). Through generation of an extensive mutagenesis library, Laencina et al. associated disruptions of the ESX-4 locus with a decrease in intramacrophage and amoeba survival. ESX-4 associated ATPase EccB4 was shown to contribute to virulence by limiting phagosomal acidification and facilitating cytosol contact through phagosomal membrane damage. Unlike ESX-3 and ESX-4, ESX-P is a plasmid-borne secretion system unique to a clinical isolate of *M. bolletti* (Dumas et al., 2016). More recent work has characterized plasmid-borne ESX components within *M. abscessus* clinical isolates, suggesting a more extensive presence of ESX systems in this species than previously thought (Dedrick et al., 2021). Whether these secretion systems are functional and contribute to *M. abscessus* virulence is an area of continued research, and may represent an unexplored means by which *M. abscessus* is able to acquire novel VFs that contribute to evolving pathogenicity.

### Lsr2

Lsr2 is a histone-like protein possessed by several members of the *Mycobacterium* genus, with roles in transcriptional regulation and DNA damage protection. In *M. tb*, Lsr2 is required for normal growth in anaerobic and DNA damage-inducing conditions (Bartek et al., 2014). When functioning as a transcriptional regulator, the binding of Lsr2 to coding sequences (of which VF genes are common) may contribute to the transition to a latent metabolic state that occurs during chronic *M. tb* infection (Gordon et al., 2010; Bartek et al., 2014). In *M. smegmatis*, *lsr2*-knockout strains have decreased persistence within macrophages, an effect which is reversed following scavenger treatment to sequester free radicals (Colangeli et al., 2009). *Mycobacterium smegmatis lsr2* knockouts also display a smooth, glossy phenotype and are defective in biofilm and pellicle formation (Chen et al., 2006). This is reflected in *M. abscessus*, where rough morphotypes display greater levels of Lsr2 expression than smooth morphotypes. While *M. abscessus lsr2* knockouts are not affected in their glycopeptidolipids (GPL) profile, *lsr2* knockout in the rough morphotype of *M. abscessus* show greater susceptibility to reactive oxygen intermediates and display reduced survival within macrophages, as well as in the zebrafish and murine model of infection, particularly at later time points during infection (Le Moigne et al., 2019). Given the importance of this transcriptional regulator for pathogenesis of *M. abscessus*, Lsr2 may be a promising target for drug development (Liu and Gordon, 2012).

### Glycopeptidolipids

*Mycobacterium abscessus* grown on solid agar medium displays distinct colony morphologies characterized as smooth (S) and rough (R) morphotypes, each with unique patterns of virulence. These are linked to the variable expression of GPL on the cell surface of *M. abscessus*, with expression of GPL within S morphotypes resulting in round, glossy colonies, while absence of GPL leads to R morphotypes resulting in a dry, corded appearance (Nessar et al., 2011). The localization of *M. abscessus* GPL has been shown to cluster to specific nanodomains on the bacterial surface, ultimately modulating surface hydrophobicity and likely influencing bacterial adhesion and virulence (Nessar et al., 2011; Viljoen et al., 2020). The conversion of S to R occur as a result of mutation within genes located within the GPL locus, often associated with GPL synthesis or secretion (Nessar et al., 2011; Pawlik et al., 2013; Park et al., 2015; Li et al., 2020). To date, the mechanisms responsible for S to R conversion are not known; however, this has been hypothesized to occur in response to stress (e.g., Antibiotic stress) which may manifest during host colonization (Bernut et al., 2016b; Gutiérrez et al., 2018). R morphotypes are more frequently isolated from CF patients with chronic infection, and display heightened virulence compared to smooth morphotypes (Jönsson et al., 2007; Kreutzfeldt et al., 2013). The differences in virulence can be linked to different interactions between S and R morphotypes with immune subsets following infection.

As surface molecules on *M. abscessus*, the absence of GPL on the surface of R morphotypes alters the initial interaction between bacteria and phagocytic cells. A lack of surface GPL exposes immunostimulatory ligands such phosphatidyl-inositol mannoside (PIM) moieties which activate Toll-like receptor (TLR) signaling, resulting in downstream pro-inflammatory cytokine production (Rhoades et al., 2009). While S morphotypes also express PIMs, the heightened expression of surface GPL masks these TLR ligands which acts to prevent innate cell activation (Davidson et al., 2011). Polar GPL themselves also act in an immunosuppressive manner, dampening pro-apoptotic signals induced by R morphotypes and limiting reactive oxygen species (ROS) production (Whang et al., 2017). It is likely that GPL also play an important role in cell–cell interactions, with post-translational modifications of GPL affecting adherence and invasion of *M. abscessus* to macrophages (Daher et al., 2020). Remodeling of surface GPL also occurs following growth in artificial CF sputum, although the biological significance of this is unknown (Wiersma et al., 2020). Together, these data point to the prominent role of GPL in defining early infection outcomes, with immunologically “silent” establishment of S morphotype *M. abscessus* in the upper respiratory tract likely facilitating initial colonization of the lung (Gutiérrez et al., 2018).

The S and R morphologies of *M. abscessus* interact differently with immune cells, leading to different patterns of growth *in vivo*. While S morphotypes exist largely as single bacilli, R morphotypes form clumps which are difficult for phagocytic cells to engulf (Roux et al., 2016). These aggregates remain adhered to phagocytic cups or, if small enough, are contained within “social” phagosomes containing multiple bacilli. Within the macrophage, R morphotypes appear resistant to lysosomal degradation, leading to extensive and rapid intracellular growth, followed by macrophage apoptosis (Aguilo et al., 2013; Kim et al., 2019). Following apoptosis, rough bacilli are then released into the extracellular space whereby they replicate freely and aggregate to form large extracellular serpentine cords which resist phagocytosis, causing excessive inflammation and abscess formation (Bernut et al., 2014a). Disruption of *M. abscessus* cording attenuates virulence *in vivo*, indicating the importance of this phenotype in dictating the outcome of infection (Bernut et al., 2014a; Halloum et al., 2016).

S morphotypes of *M. abscessus* also resist intracellular degradation, through more direct manipulation of macrophage effector function. Phagocytosed S bacilli often exist in “loner phagosomes,” and act to limit phagosomal acidification and disrupt phagosome membrane integrity, which may facilitate escape to the cytosol (Roux et al., 2016). While cytosol escape and blocking of phagosome acidification are associated with functional ESX-4 found in *M. abscessus*, it is unclear what role surface GPL may play in facilitating this effector function (Gutiérrez et al., 2018; Laencina et al., 2018). Given the integral link between mycobacterial virulence and macrophage function, understanding the mechanisms by which *M. abscessus* interacts with innate immune cells is critical in our understanding of *M. abscessus* virulence (Feng et al., 2020).

### Other Cell Surface Molecules

The structure of the mycobacterial cell wall and outer envelope is comprised of a complex array of lipoproteins, glycolipids, and glycoproteins, many of which take part in host–pathogen interactions (Jackson, 2014). These may directly modulate immune responses, or improve the structural integrity of the mycomembrane which renders *M. abscessus* more resistant to immune mediators (Karakousis et al., 2004). For example, succinylation of *M. abscessus* surface polysaccharides alters intracellular survival by an uncharacterized mechanism, possibly in relation to altered cell surface hydrophobicity and charge (Palčeková et al., 2020). Other VFs may arise from proteins associated with the modification or transport of cell surface molecules. Knockout of *pmt*, a protein-O-mannosyltransferase which is responsible for glycosylation of lipoproteins in the mycobacterial cell envelope, increases cell wall permeability which may enhance susceptibility to innate cellular defenses, such as ROS (Becker et al., 2017). Similarly, probable N-acetyl transferase Eis2 is thought to play a role in cell wall biogenesis or transport of cell wall components; deletion of this VF increases *M. abscessus* susceptibility to ROS and H_2_O_2_ and reduces intracellular survival (Dubois et al., 2019). Expression of some surface components, such as trehalose polyphelates (TPPs), does not directly affect macrophage viability, but promote virulence by facilitating *M. abscessus* cording (Llorens-Fons et al., 2017). *Mycobacterium abscessus* cell envelope-derived lipids, such as cardiolipin or I-mannosides, are also effective at neutralizing LL-37, an antimicrobial peptide produced by neutrophils and macrophages (Honda et al., 2015, 2020). It has been established that different growth conditions alters the cell wall composition of *M. abscessus*; whether this occurs in an infection setting and how this impacts on the outcome of infection remains to be established (Hunt-Serracin et al., 2019).

## Non-mycobacteria-Specific Virulence Factors

**Figure 1:**
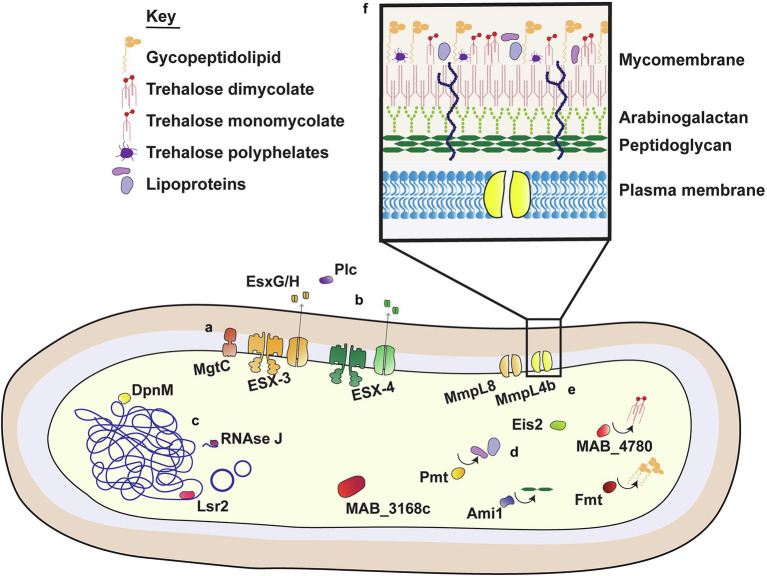
Virulence factors of *M. abscessus*. M. abscessus uses an extensive range of virulence factors to facilitate survival within host cells. These include membrane bound proteins, pores and secretion systems (i); secreted proteins with a role in virulence (ii); transcriptional regulators and nucleic acid associated proteins (iii); proteins involved in the modification (iv) and transport (v) of cell surface components; and molecules that comprise the outer mycobacterial membrane (vi).

While *M. abscessus* was first isolated in 1952, complete sequencing of the *M. abscessus* genome was completed relatively recently in 2009 (Brown-Elliott and Wallace, 2002; Ripoll et al., 2009). In their genomic analysis of *M. abscessus*, Ripoll et al. identified regions syntenic with non-mycobacterial species, including Actinobacteria, such as *Rhodococcus*, as well as opportunistic CF pathogens *P. aeruginosa* and *Burkholderia cepacea* (Ripoll et al., 2009; Mahenthiralingam, 2014). The organization of these genes into large clusters was suggestive of *en bloc* horizontal gene transfer events from distant species, with some of these regions encoding proteins that are known to promote virulence in other species. The functions of these VFs are well defined in other species, with most facilitating survival in harsh or stressful conditions. Some are involved in early *P. aeruginosa* colonization and/or persistence in CF airways, such as phenazine biosynthesis and homogentisate catabolism genes (Rodriguez-Rojas et al., 2009; Jimenez et al., 2012). VFs involved in iron acquisition and resistance to reactive nitrogen intermediates (RNI) were also identified, with sequence homology to *Rhodococcus* spp. (Ripoll et al., 2009). Similarly, the *M. abscessus* genome contained proteins with predicted function in phenylacetic acid degradation; these promote *B. cenocepacia* survival in the *Caenorhabditis elegans* models of infection, although their precise mechanism of action is unknown (Law et al., 2008; Loutet and Valvano, 2010). Some of these non-mycobacterial VFs have been examined in detail (Figure 1), however for the most part their function in *M. abscessus* virulence is yet to be established.

### Phospholipase C

Phospholipases are virulence determinants found in a diverse range of mycobacterial and non-mycobacterial species, including *P. aeruginosa*, *Staphylococcus aureus*, and *Listeria monocytogenes* (Ghannoum, 2000). Phospholipases promote virulence by allowing escape from the phagosome and cell-to-cell spread, which is thought to be mediated by cleavage of phospholipids that form part of the cell membrane (Camilli et al., 1991; Smith et al., 1995; Ghannoum, 2000). Expression of *plc* genes by *P. aeruginosa* is associated with phosphate limiting conditions, and disruption of *plcC* results in a reduction of *P. aeruginosa* virulence in the murine model of infection (Ostroff et al., 1989). Similarly, phospholipase C is upregulated by virulent *M. tb* in phosphate limiting conditions, such as those that may occur in an intracellular environment (Le Chevalier et al., 2015). PlcC isolated from *M. abscessus* induces macrophage lysis, and *plcC* knockout strains display reduced survival within amoeba (N’Goma et al., 2015). Given the sequence similarity between *plcC* from *M. abscessus* and other CF pathogens (N’Goma et al., 2015), PlcC is a promising antigenic target for vaccine development. CF patients with pulmonary NTM infection or *P. aeruginosa* infection have higher titers of anti-PlcC antibodies in serum than uninfected individuals, indicating immune exposure to the PlcC antigen following infection (Le Moigne et al., 2015). Vaccination with *M. abscessus plcC* DNA induces potent antibody responses and a moderate reduction in bacterial burden in ΔF508 mice, which possess the most common CF gene mutation (Cutting, 2015; Le Moigne et al., 2015). However, the role of PlcC in nutrient acquisition in the lung, and if it enables *M. abscessus* to maintain long-term infection is unclear (Raynaud et al., 2002).

### MgtC

MgtC plays an essential role in the survival of pathogens such *Salmonella enterica* subsp. *typhimurium* and CF pathogens *B. cenopacea* and *P. aeruginosa* (Alix and Blanc-Potard, 2007; Belon et al., 2015). While the physiological function of this protein has not been fully elucidated, MgtC is a membrane protein that confers survival benefits for both intracellular and extracellular pathogens (Belon et al., 2015). Expression is essential for growth in magnesium poor conditions for *P. aeruginosa* and *B. cenopacea*, while upregulation of MgtC following phagocytosis provides enhanced intracellular survival and resistance to low pH for intracellular pathogen *Salmonella* spp. (Maloney and Valvano, 2006; Rang et al., 2007; Lee and Lee, 2015). MgtC appears to perform a similar role in *M. tb*, as the protein contributes to improved survival in low magnesium and pH conditions and enhanced virulence in mice (Buchmeier et al., 2000). *Mycobacterium abscessus* has two sequences encoding proteins homologous to MgtC, MAB_3953, and MAB_0146, both of which bear sufficient sequence similarity to *S. typhurmium* MgtC, as they can partially restore its function in an *mgtC*-deficient strain (Le Moigne et al., 2016). *Mycobacterium abscessus* MgtC is induced in low Mg^2+^ conditions and within the macrophage, *mgtC-*encoding DNA provides protection against *M. abscessus* challenge (Le Moigne et al., 2015, 2016). Of note, *M. abscessus* lacking functional MgtC does not show a significant impairment in macrophage survival, which may indicate a role of this VF in the extracellular stage of *M. abscessus* growth (Bernut et al., 2014a).

### Porins

Porins are a diverse class of pore proteins that play an important role in the transport of hydrophilic molecules into the bacterial cell. Porins are expressed widely among gram-positive and negative virulence of different species (Achouak et al., 2001). The contribution of porins to the virulence of different species of pathogenic bacteria is extremely diverse and includes facilitating cell adherence, inducing host cell apoptosis and transporting surface proteins associated with virulence (Müller et al., 1999; Azghani et al., 2002; Brunson et al., 2019). Within the context of mycobacteria, *M. smegmatis* has a collection of pore-forming proteins that regulate nutrient influx which is important for maintaining normal growth rates (Stephan et al., 2005). Deletion of *M. smegmatis* porin MspA, MspC, and MspD improves *M. smegmatis* survival in macrophages, by providing greater resistance of *M. smegmatis* to host-produced nitric oxide (NO; Fabrino et al., 2009). Similarly, deletion of porins mmpA/B in *M. abscessus* also improves *M. abscessus* intracellular survival within phagocytic cells and bacterial persistence in SCID mice (de Moura et al., 2021). Porin knockout strain Δ*mmpA* displays reduced uptake of glucose, but mutants did not display varied uptake by macrophages, susceptibility to NO, or cell cytotoxicity. Of note, sequential samples taken from different early and late *M. abscessus* infections in CF patients show mutations within the *mmp* porin genes (Lewin et al., 2021). However, phylogenetic analysis by de Moura et al. could not establish a contributing role of porin mutations to enhanced virulence or transmission of dominant clinical *M. abscessus* isolates (de Moura et al., 2021). This suggests that while this class of proteins are significant VFs of other species, porins possessed by mycobacterial species including *M. abscessus* may perform a role unrelated to virulence.

## Toward the Development of a *Mycobacterium abscessus* Vaccine: Possibilities and Challenges

The global incidence of NTM including *M. abscessus* is increasing, with disease prevalence within CF populations increasing from 9% to 13% in studies conducted after 2000 (Marras et al., 2007; Qvist et al., 2014, 2015; Bar-On et al., 2015; Adjemian et al., 2018). Chronic infection with *M. abscessus* leads to decreased lung function and is a significant contributor to morbidity and mortality of affected individuals (Esther et al., 2010; Qvist et al., 2015). This combined with the extensive antibiotic resistance and large repertoire of VFs of this pathogen (Figure 1; Table 1) emphasizes the need for prophylactic strategies to limit the global burden of *M. abscessus*. Given there is currently no vaccine available for *M. abscessus* and none in clinical development, there is a clear unmet need for the development of vaccines against this pathogen. However, development of a potential *M. abscessus* vaccine must consider target populations for vaccination, and specific challenges associated with immunizing these populations. This includes an understanding of immune correlates of protection against *M. abscessus* in both immunocompetent and immunocompromised individuals, in addition to gaining knowledge from efforts to develop a vaccine for *M. tb*.

**Table 1:** Known virulence factors of *Mycobacterium abscessus.*

Name of virulence factor	Corresponding gene in reference strain	Distribution and biological function	Role of virulence factor in *M. abscessus*	Immunogenicity and/or protective efficacy	References
MgtC	MAB_3953	Membrane bound ATPase, found in a range of CF and non-CF pathogens including *Salmonella enterica*, *Burkholderia cenocepacea* and *M. tb*	Required for optimal growth in magnesium poor media; upregulated upon intracellular macrophage infection	Ma-MgtC specific antibodies present within the serum of *M. abscessus* positive CF patients. DNA vaccination with MgtC plasmid reduces bacterial burden in ΔF508 CFTR mice	Belon and Blanc-Potard, 2016; Le Moigne et al., 2016
PlcC	MAB_0555	Phospholipase involved in virulence of numerous bacterial pathogens including *P. aeruginosa*, *L. monocytogenes*	Induces eukaryotic cell lysis and promotes *M. abscessus* intracellular survival in *A. castellanni* amoeba	DNA vaccination with *M. abscessus*-PLC induces anti-PLC antibodies and significantly reduces bacterial burden in lungs of ΔF508 mice. Presence of anti-PLC antibodies in CF patients with *M. abscessus* and *P. aeruginosa* infection	Le Moigne et al., 2015; N’Goma et al., 2015
eccB4 (component of ESX-4)	MAB_3759c	Membrane component of the ESX-4 locus	Promotes *M. abscessus* survival within amoeba and macrophages by inhibition of phagosome acidification and promoting phagosome-cytosol contact	Induces IL-1β production by facilitating cytosolic contact	Laencina et al., 2018
ESX-3	MAB_2224c-MAB_2234c	ESX protein secretion system	Improves *M. abscessus* survival in the animal model of infection	ΔESX-3 causes reduced inflammatory cytokine production by macrophages, reduced cell infiltration to the lungs and production of COX2 and iNOS and impaired NFκB activation in macrophages	Kim et al., 2017
MAB_4780	MAB_4780	Dehydratase, possibly involved in mycolic acid metabolism	Provides resistance to anti tubercular drug thiacetazone. Essential for extracellular cording of rough *M. abscessus*; involved in intracellular survival and granuloma formation within zebrafish model of infection	Not determined	Halloum et al., 2016
Lsr2	MAB_0545	Nucleoid associated protein/ transcriptional regulator that binds AT-rich genomic regions. Present in other mycobacterial species and essential for growth of *M. tb*	Expressed at higher levels in rough variants; Absence of Lsr2 in R variants increases susceptibility to reactive oxides and reduces intracellular survival in *A. castellani* and macrophages. Enhances virulence in zebrafish and bacterial persistence in mice	Not determined	Le Moigne et al., 2019
MmpL4b	MAB_4115c	Membrane bound protein involved in facilitating gylcopeptidolipid transport to *M. abscessus* surface	Disruption of mmpL4b results in S to R transitioning, enhanced extracellular cord and abscess formation in the zebrafish model of protection. *ΔmmpL4b M. abscessus* also displays enhanced replication in macrophages	ΔmmpL4b S mutants induce TLR stimulation and production of TNF	Medjahed and Reyrat, 2009; Nessar et al., 2011; Bernut et al., 2016b
MmpL8_MAB_	MAB_0855	Large membrane permease involved in transport of glycolipids through the plasma membrane	Promotes intracellular survival and adherence to macrophages in S colony morphotype; mutants retain ability to cause phagosomal acidification but reduced ability to establish cytosolic contact	Induces IL-1β production by facilitating phagosome-cytosol contact	Dubois et al., 2018
Pmt	MAB_1122c	Protein-O-mannosyltransferase responsible for glycosylation of lipoproteins in the mycobacterial cell envelope	*Δpmt* display increased antibiotic susceptibility to β-lactams and high molecular weight antibiotics, increased cell wall permeability and decreased intracellular survival	Not determined	Becker et al., 2017
Fmt	Not determined	Fatty acid O-methyltransferase responsible for methoxylation of fatty acyl chain of GPL	*Δfmt* displays reduced cell surface hydrophobicity and enhanced adherence to and invasion of THP-1 macrophages, but no change in intracellular survival	Not determined	Daher et al., 2020
MAB_3168c	MAB_3168c	Acetyltransferase	Defect in MAB_3168c results in transition from R to S morphotype, increased susceptibility to lysozyme and amikacin and reduced intracellular survival in macrophages	Not determined	Tsai et al., 2013
GPL	Various	Surface glycopeptidolipids	Loss of GPL on *M. abscessus* surface causes smooth to rough transition; rough morphotype associated with increased virulence and inflammation.	GPL isolated from S morphotypes limit apoptosis, ROS production and cytochrome C release in macrophages. Surface bound GPL also limits TLR activation	Rhoades et al., 2009; Davidson et al., 2011; Whang et al., 2017; Gutiérrez et al., 2018
Polar mycobacterial lipids	Various	Secreted and/or surface bound	LL-37 (cathelicidin) loses antimicrobial activity when pre-incubated with *M. abscessus* derived polar mycobacterial lipids	Not determined	Honda et al., 2015
Eis2	MAB_4532c	N-acetyl transferase, similarity to MmpL11 locus in *M. tb* with potential role in cell wall biogenesis	Δ*Eis2* has reduced intracellular survival, facilitates phagosome-cytosol escape, and shows greater sensitivity to ROS and H_2_O_2_	Not determined	Dubois et al., 2019
MAB_2560	MAB_2560	Not determined	Not determined	Induces DC maturation and co-stimulatory molecule expression in a TLR4 dependent manner and through MAPK mediated signaling; MAB_2560 stimulated DCs induce T-cell maturation and Th1 polarization with OVA antigen	Lee et al., 2014
Ami1	MAB_0318c	N-acetylmuramyl-L-alanine amidase involved in the remodeling of peptidoglycans on mycobacterial surface	Overexpression of Ami1 enhances survival within THP-1 macrophages; further supplementation promotes this effect. Not required for virulence in zebrafish.	Not determined	Küssau et al., 2020
RNAse J	MAB_3083c	Gene encoding RNAse J homologue, involved in mRNA metabolism—ribosomal maturation and mRNA stability	Knockout is involved in smooth to rough conversion, with MAB_3083c∷*Tn* displaying increased sliding motility and decreased aggregation; complementation causes reversion back to the rough form. Knockout does not have any effect on intracellular growth, H_2_O_2_ or lysozyme susceptibility	Not determined	Liu et al., 2021
DpnM	Not determined	DNA methyltransferase	Knockout has differing expression of genes involved in stress response and intramacrophage survival; knockouts also display enhanced susceptibility to NO and amikacin, and reduced intracellular survival	Not determined	Bryant et al., 2021
mmpA, mmpB	MAB_1080,MAB_1081	Porin involved in transport across cell membrane	Deletion of MmpA/B enhances virulence of *M. abscessus* in macrophages and SCID mice, does not affect cell cytotoxicity or macrophage uptake	Not determined	de Moura et al., 2021

### Immune Responses to *Mycobacterium abscessus* Infection

A thorough understanding of the immune correlates of protection against *M. abscessus* infection is critical for the development of an effective *M. abscessus* vaccine. This is an area of ongoing research, primarily focused on the use of *in vitro*, zebrafish, and murine models (Bernut et al., 2015, 2017; Caverly et al., 2015; Meir et al., 2018). Incorporating these different approaches has led to a greater knowledge of immune mechanisms involved in protection against this pathogen.

As with other members of the *Mycobacterium* genus, early and robust innate immune responses appear to play a critical role in shaping immune control of *M. abscessus* infection. Monocytes and macrophages are the most common cell subset infected by *M. abscessus* in human lung tissue (Ganbat et al., 2016). Both S and R morphotypes of *M. abscessus* are rapidly phagocytosed by macrophages early after infection and depletion of this subset substantially increases bacterial burden in zebrafish (Bernut et al., 2014a 2019). ROS and NO are major bactericidal mechanisms employed by macrophages to kill *M. abscessus*, with production of NO and ROS strongly correlated with a protective effect across *in vitro* and animal models of *M. abscessus* infection (Kim et al., 2014a; Lee et al., 2017). Interestingly, NO production is enhanced by a type I interferon (TI IFN) response and prophylactic administration of rIFN-Β can promote bacterial clearance (Lee et al., 2017). This is contrary to recent work on *M. abscessus* suggesting that TI IFNs promote macrophage apoptosis and bacterial spread from cell-to-cell (Zhang et al., 2019). In humans, the role of NO and ROS in providing protection against *M. abscessus* is less clear. When used as an inhaled therapeutic, NO improves lung function and quality of life in CF patients, but this has not been definitively linked to reduction in *M. abscessus* burden (Bentur et al., 2020). An oxidative environment within the macrophage also appears to enhance *M. abscessus* growth in an *ex vivo* setting (Oberley-Deegan et al., 2010). Moreover, NOS2 knockout (*Nos2^−/−^*) mice do not display exacerbated bacterial burden compared to wild type (Obregón-Henao et al., 2015). Taken together, these findings suggest a redundant or possibly detrimental role of ROS in protection against *M. abscessus* infection.

As early responders to pulmonary infection, neutrophils are a prominent component of the immune response against *M. abscessus*. Neutrophil accumulation is driven by TNF and IL-8 production by macrophages and alveolar epithelial cells, and this subset congregates within murine lungs following infection with both S and R morphotypes (Caverly et al., 2015; Bernut et al., 2016a; Malcolm et al., 2018). Despite the integral role of neutrophils in the formation of the granuloma, the efficacy of this subset in reducing bacterial burden remains unclear. Neutrophils phagocytose bacteria and employ neutrophil extracellular traps (NETs) and ROS production to eliminate *M. abscessus*, but neutrophil-derived ROS and LL-37 has limited killing activity against *M. abscessus* (Honda et al., 2015; Malcolm et al., 2018). Extracellular DNA released in response to *M. abscessus* during NETosis may also contribute to biofilm formation in the CF lung (Malcolm et al., 2013). Pulmonary infection with the more virulent R morphotype of *M. abscessus* is also accompanied by neutrophil accumulation in the bronchoalveolar lavage fluid, to a greater extent than that induced by the S morphotype (Caverly et al., 2015). Given the inflammatory nature of this immune cell subset, excessive neutrophilia can be extremely damaging to lung tissue and is a primary cause of tissue damage in CF (Downey et al., 2009). How exactly this subset contributes to protection or pathology following chronic infection is an area of continued research.

**Figure 2:**
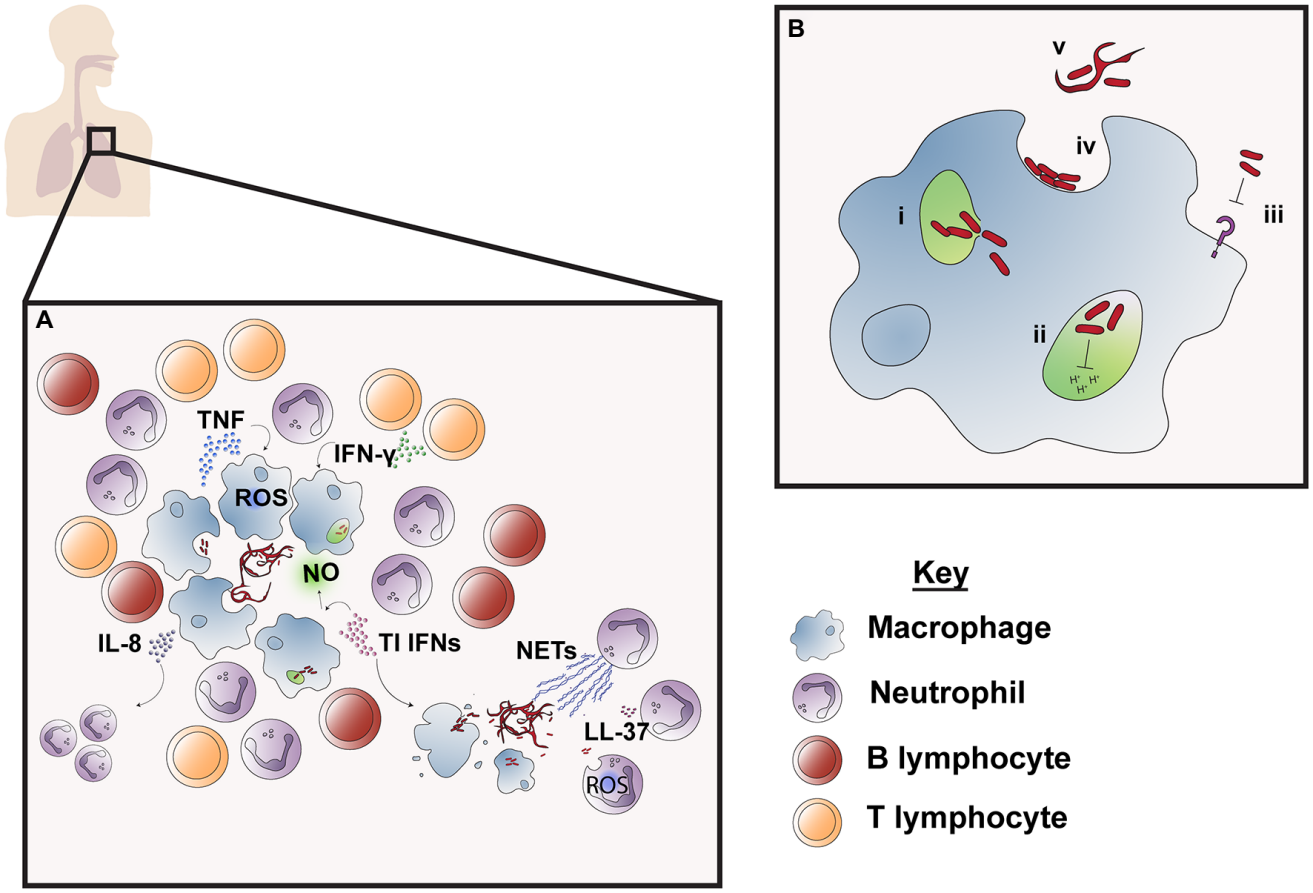
Immune responses to *M. abscessus* infection*. M. abscessus* infection is followed by an influx of neutrophils and macrophages surrounded by lymphocytes which work to contain bacteria in the granuloma **(A)**. Cytokines TNF and IFN-γ produced by macrophages and CD4^+^ T cells are required for granuloma formation and induce macrophage effector function, such as phagosome acidification and reactive oxygen species (ROS) production, while type I IFN production promotes nitric oxide (NO) production. IL-8 attracts neutrophils which are responsible for phagocytosis, NET production and secretion of antimicrobial peptide LL-37. However, release of *M. abscessus* into the extracellular space as a result of cell death leads to the formation of serpentine cords, which are resistant to innate immune defenses and leads to unchecked bacterial replication. *M. abscessus* also possesses numerous mechanisms of immune evasion to resist macrophage effector functions **(B)**; these include bacterial escape from the phagosome to the cytosol through interference with the phagosomal membrane (i); prolonged survival within the phagosome by blocking phagosomal acidification and thereby preventing *M. abscessus* degradation (ii) and inhibition of macrophage TLR signaling which limits downstream immune cell activation and recruitment (iii). *M. abscessus* also persists in the extracellular environment by avoiding phagocytosis, which is enabled through adherence to macrophage phagocytic cups on the cell surface (iv) and by forming serpentine cords which are too large to be engulfed by macrophages (v).

A cornerstone of the immune response to mycobacterial infection is the formation of the granuloma, a cluster of recruited host immune cell subsets which form a physical structure to restrict bacteria (Figure 2A). In zebrafish, *M. abscessus* granulomas are primarily comprised of neutrophils and macrophages. The accumulation of these subsets is dependent on TNF, which is required for development of the granuloma structure and the containment of bacterial growth (Bernut et al., 2016a). However, granuloma formation is not always sufficient in containing *M. abscessus* infection—the induction of macrophage cell death by *M. abscessus* releases extracellular bacteria, which form large serpentine cords that evade phagocytosis and resist immune defenses (Figure 2B; Bernut et al., 2014a; Halloum et al., 2016). Currently, the specific immunological factors differentiating protective granuloma formation from unrestrained bacterial growth are not known (Johansen et al., 2020). Murine models of *M. abscessus* infection also display granuloma formation in the lungs and spleen after aerosol or intravenous infection, respectively, and studies using TNF knockout (*Tnf^−/−^*) and IFN-γ knockout (*Ifngr1*^−/−^ and GKO^−/−^) mice highlight the importance of these cytokines for maintenance of granuloma structure (Rottman et al., 2007; Ordway et al., 2008). Nude, severe combined immunodeficiency (SCID) and GMCSF knockout (GMCSF^−/−^) mice also develop progressive granulomatous lesions, making these useful models for preclinical drug testing but less suitable for understanding immune factors involved in granuloma formation (Obregón-Henao et al., 2015). While evidence from animal models points to a significant role of this structure in control of *M. abscessus* infection, the importance of granuloma formation in humans is less clear. Granuloma formation is well documented following cutaneous *M. abscessus* infection (Bartralot et al., 2000; Kwon et al., 2009), but fewer studies characterize the granulomatous response following pulmonary infection (Jeong et al., 2004; Okazaki et al., 2013). *Mycobacterium abscessus* results in heterogeneous changes to pulmonary tissue following infection, including inflammatory infiltrate which may be accompanied by the formation of necrotizing or non-necrotizing granulomas (Jeong et al., 2004). Based on what is known about other NTM infections, host immune responses, clinical isolate heterogeneity, and pathogen virulence may all contribute to the extent of granuloma formation and its ability to contain *M. abscessus* infection (Lammas et al., 2002).

Most relevant for vaccine design is the contribution of adaptive immune subsets to *M. abscessus* pulmonary infection. CD4^+^ T helper (T_H_) cells appear to be a key component of the protective response, with T_H_1 responses enabling control of mycobacterial dissemination and granuloma formation through their stimulation of innate immune subsets, such as macrophages and neutrophils (Rottman et al., 2007; Bernut et al., 2016a). In a murine model of *M. abscessus* infection, bacterial clearance is preceded by an influx of IFN-γ producing CD4^+^ T cells, and it has been shown that *M. abscessus* infection persists in IFN-γ knockout GKO^−/−^ mice (Ordway et al., 2008; Obregón-Henao et al., 2015). High-dose infection models in GKO^−/−^ mice display a heightened T_H_2 response resulting in an immunosuppressive phenotype, supporting the notion that T_H_1 polarization is ideal for protection against intracellular mycobacteria (Ordway et al., 2008). Similarly, individuals with deficiencies affecting T-cell function appear predisposed to *M. abscessus* infection (Lutzky et al., 2018). Patients with active *M. abscessus* infection also display a dampened T_H_1/T_H_2 and heightened T_H_17 cytokine profile compared to healthy controls, suggestive of a link between T_H_1 polarization and a protective phenotype (Kim et al., 2014b; Lake et al., 2016). This is an especially important consideration in the design of subunit *M. abscessus* vaccines, where adjuvant choice affects T-cell polarization and thus resulting protection (Knudsen et al., 2016).

While robust T-cell responses are clearly essential for protection against *M. abscessus*, the contribution of the humoral response to protection against pulmonary *M. abscessus* infection is less established. Antibodies to *M. abscessus* are generated following infection or vaccination (Jeon et al., 2009; Le Moigne et al., 2015) and B-cell deficiency in the murine model of infection promotes bacterial growth (Rottman et al., 2007). While humoral responses in healthy individuals are considered a critical components for extracellular pathogens, such as *P. aeruginosa*, their efficacy appears limited in preventing bacterial colonization or eradicating infection in CF patients (Yonker et al., 2015; Sainz-Mejías et al., 2020). Humoral responses that target surface GPL of *M. abscessus* S morphotypes may also encourage the conversion to the more virulent R morphotype, thus proving detrimental to the host (Gutiérrez et al., 2018). It is also unclear whether the extracellular stages of *M. abscessus* growth, such as biofilm formation during early infection, and extracellular cording following phagosome rupture would be effectively disrupted by neutralizing antibodies. Most vaccines currently approved for human use focus on the effective development of humoral responses, and development of vaccines targeting cell-mediated immunity appear more difficult to produce. As such, the contribution of this subset to *M. abscessus* protection will require continued investigation to inform development of an effective *M. abscessus* vaccine.

### The Quest for a *Mycobacterium abscessus* Vaccine in Susceptible Populations

*Mycobacterium abscessus* infection primarily occurs in individuals with reduced pulmonary immune responses, including those with CF, COPD, and non-CF bronchiectasis (Ratnatunga et al., 2020). The development of novel immunization strategies for this pathogen has the potential to dramatically reduce the *M. abscessus* burden and improve quality of life for these susceptible populations. However, progress toward novel vaccines has been limited by an incomplete understanding of immune responses in these groups that perpetuate *M. abscessus* infection, and how this may impact the outcome of vaccination. While populations with COPD and non-CF bronchiectasis make up a significant proportion of *M. abscessus* cases, factors resulting in enhanced susceptibility of these populations to NTM infection have not yet been established (Griffith et al., 2007; Ratnatunga et al., 2020). It is speculated that structural damage within the lung in non-CF bronchiectasis permits bacterial colonization, but immune dysfunction, such as aberrant neutrophil migration and effector function, also perpetuates infection by preventing optimal clearance (Chalmers and Hill, 2013). Populations susceptible to NTM infection with no clear risk factors display reduced IFN in serum and altered adipokine levels, further suggesting an underlying link between immune responses and risk of infection (Kartalija et al., 2013).

Given the frequency with which CF populations become infected with NTM, the consideration of immune responses in CF populations is of utmost importance when developing a vaccine for *M. abscessus*. As research into the area continues, we are beginning to appreciate the profound impact of CFTR mutations within CF patients on immune cell functionality, and the implications of this for pulmonary bacterial clearance (Hartl et al., 2012). The immune landscape of CF patients includes macrophages with a skewed hyperinflammatory profile (Bruscia and Bonfield, 2016), neutrophils with dysfunctional phagosomal maturation and effector functions (Zhou et al., 2013; Gifford and Chalmers, 2014), and an inflammatory milieu that may limit the ability of innate cells to clear infection (Roghanian et al., 2006; Cockx et al., 2018). Recent work by Bernut et al. also showed impaired ROS production in professional phagocytic cells following *M. abscessus* infection, using a zebrafish model of CF (Bernut et al., 2019). While less is known about the implications of CFTR mutations on adaptive immune responses, lymphocytes from murine models of CF also display a predilection to the development of T_H_2 and T_H_17 responses (Mueller et al., 2011; Tiringer et al., 2013; Mulcahy et al., 2015). *Cftr^−/−^* mice also display dysregulated B-cell follicle formation, activation, and accumulation in the lung (Polverino et al., 2019). This adds to the complexity of vaccine development for *M. abscessus*, as immune responses may be harder to predict in target populations and could potentially be naturally skewed toward non-protective phenotypes (Kartalija et al., 2013). The quality of immune responses within these populations will play a significant role in shaping the outcome of vaccination with an *M. abscessus* vaccine and is therefore a key area of continued research.

Research of potential therapeutic agents to protect against *M. abscessus* infection may greatly benefit from effective animal models that recapitulate the clinical presentation of *M. abscessus* lung disease. This has been a continual challenge for researchers, as animal genotypes that possess common CF mutations do not display the same pathological symptoms as humans (Guilbault et al., 2007). For example, the ΔF508 mouse model possesses the most common mutation in the CFTR gene in humans but does not recapitulate the disease pathophysiology (Scholte et al., 2004). Although some knockout strains show greater persistence of bacterial load compared to wild-type littermates, none of these models develop spontaneous colonization or persistent infection by CF pathogens in the lung, such as in human patients (McMorran et al., 2001; Guilbault et al., 2005, 2007; Ordway et al., 2008; Bernut et al., 2014b). This problem has been circumvented by the use of immunocompromised strains (Lerat et al., 2014), corticosteroid administration (Maggioncalda et al., 2020), or infection with thrombin and fibrinogen plugs; a strategy similar to the agarose bead infection model used for *P. aeruginosa* (Facchini et al., 2014; Caverly et al., 2015). In addition to zebrafish and *A. castellanni* models, these have provided us with snapshots of the immune response to *M. abscessus* infection; however, an in-depth understanding of correlates of protection is best achieved with immune competent models which recapitulate the immune response to *M. abscessus* infection in humans.

The discovery of VF acquisition by *M. abscessus* from other CF pathogens highlights the continued evolutionary potential of this pathogen and reinforces the urgent need for vaccines to provide robust protection to susceptible groups. Suggestions that the CF lung provides an environment that may enhance *M. abscessus* virulence is particularly worrisome; however, shared VFs across different bacterial species could allow the development of protein vaccine candidates that target multiple CF pathogens (Ripoll et al., 2009; Bryant et al., 2021). For instance, PlcC antibodies from CF patients infected with *P. aeruginosa* are cross-reactive with *M. abscessus* PlcC (Le Moigne et al., 2015). Cross-protective vaccines are clearly advantageous in terms of research and development costs; however, no *M. abscessus* vaccine study to date has confirmed a protective effect of cross-reactive immune responses induced by vaccination (Le Moigne et al., 2016). Given the number of VFs acquired from non-mycobacterial species in the *M. abscessus* genome, this is a noteworthy area of continued research (Ripoll et al., 2009).

In addition to the development of vaccines for CF pathogens, there is interest in development of an *M. abscessus* vaccine to provide protection against different mycobacterial species. Lower incidence rates of *Mycobacterium avium* infection in HIV-positive individuals with prior *M. tb* infection suggest a degree of protection afforded by mycobacterial exposure (Horsburgh et al., 1996). This has encouraged interest in repurposing the current *M. tb* vaccine, *Mycobacterium bovis* Bacille Calmette–Guérin (BCG), to be used for protection against NTM infection in susceptible populations (World Health Organization, 2020). While lower incidences of NTM infection in BCG-vaccinated children support the notion of BCG vaccine-induced cross-protection, there is a paucity of information in the literature specifically referring to protection against *M. abscessus* (Trnka et al., 1994; Zimmermann et al., 2018). T cells isolated from peripheral blood of latent-TB-infected or BCG-vaccinated individuals produce IFN-γ and granzyme A in response to *M. avium* restimulation, and these cross-reactive T cells restrict growth of *M. avium or M. abscessus* in monocytes. BCG vaccination of mice also generates a population of T cells that secrete cytokines upon NTM restimulation; however, the effect of vaccination on bacterial burden was not determined in this study (Abate et al., 2019). As there are clear advantages of repurposing a currently approved and widely distributed vaccine, the role of BCG vaccination in providing protection against *M. abscessus* infection requires further investigation.

### Lessons Learned From TB Vaccination Efforts

Despite being heavily researched since its discovery, *M. tb* is still a leading cause of death by infectious disease worldwide. In 2020, a quarter of the world’s population was estimated to be infected with *M. tb* with 10 million new infections in 2019 alone (Chakaya et al., 2021). The protection afforded by the current vaccine available, BCG, is extremely variable across different age groups, latitudes, and to those with previous infection or mycobacterial exposure (Mangtani et al., 2014; Ahmed et al., 2021). While numerous candidates for novel TB vaccines are currently in the developmental pipeline, none have been able to provide sufficient protection to replace BCG. However, we can learn from decades of TB research and vaccine development about factors to consider in the pursuit of an *M. abscessus* vaccine, in addition to identifying unique challenges specific to this pathogen.

A robust understanding of immune responses required for protection is essential in the development of novel *M. abscessus* vaccines. In the case of vaccines against *M. tb*, efforts have been consistently stalled by our incomplete knowledge of immune correlates of protection against *M. tb* (Counoupas et al., 2019). This was exemplified in the results of the Modified Vaccinia Ankara 85A (MVA85A) vaccine in Phase IIb clinical trials, which had been a leading candidate to replace BCG. While earlier studies had shown strong T-cell-mediated immune responses following vaccination, this vaccine did not show protective efficacy in infants (Scriba et al., 2010; Tameris et al., 2013). T_H_1 helper subsets and cytokines IFN-γ and TNF also appear to be important for protection against *M. abscessus*, but whether these subsets are predictive of protection afforded by vaccination has not yet been established (Rottman et al., 2007; Bernut et al., 2016a). The difficulty in determining immune correlates of protection against *M. abscessus* is exacerbated by the diverse growth stages of this microorganism, with extracellular biofilm formation and colonization preceding the emergence of invasive intracellular variants which form chronic infection in the lung (Howard et al., 2006; Gutiérrez et al., 2018; Bryant et al., 2021). There is currently a paucity of information on how different branches of the immune response (namely, humoral and cell-mediated subsets) contribute to effective bacterial clearance across different infection stages. Diverse vaccination strategies can be aimed at preventing colonization by inducing a strong humoral response or at enhancing clearance of persistent infection by inducing a strong cell-mediated immune response. As with *M. tb* infection, it is also unclear what impact natural immunity from prior *M. abscessus* infection has on the risk of reinfection (Verver et al., 2005; McIvor et al., 2017). Recurrence of *M. abscessus* infection has been attributed to poor antimicrobial efficacy, particularly the inefficacy of macrolide treatments to fully eradicate infection (Pasipanodya et al., 2017). However, it has been noted that reinfection with NTM, such as *M. abscessus*, frequently occurs with strains of different genotype, suggesting that *M. abscessus* antigenic variability has an impact on protective immune responses (Koh et al., 2017). A comprehensive understanding of the importance of natural immunity over the duration of *M. abscessus* infection will be critical to dictate the trajectory of novel vaccine development.

#### Potential Vaccination Strategies for *Mycobacterium abscessus*

There is an enormous array of strategies that may be used in the development of a novel *M. abscessus* vaccine, each with different advantages and drawbacks. These include whole-cell (live attenuated or heat-killed), nucleic acid vaccines, and subunit (viral vectored or protein and adjuvant) vaccines.

Live attenuated vaccines have an enormous advantage in that they possess an extremely diverse repertoire of proteins that may be recognized by the immune system, thereby inducing a more diverse immune response. Beyond attenuation, whole-cell vaccines may also be modified to further enhance antigenic visibility to the immune system. VPM1002, for example, is a modified form of the BCG vaccine containing protein Listeriolysin O, which allows BCG escape into the cytosol (Grode et al., 2013). This facilitates antigen presentation to CD8^+^ T cells, thereby activating an additional component of the immune response to the vaccine (Grode et al., 2005). However, live attenuated vaccines pose an inherent risk of vaccine-associated disease for immunocompromised individuals or those on immunosuppressants, such as individuals with CF (Burroughs and Moscona, 2000; Malfroot et al., 2005). Heat-killed whole-cell vaccines are advantageous for these populations because there is no risk of vaccine dissemination and have been particularly successful in the TB field. One such example is Vaccae™, a whole-cell vaccine of inactivated environmental mycobacteria *Mycobacterium vaccae* (Fatima et al., 2020). Given the shared epitopes of *M. vaccae* with NTM and *M. tb*, this is a potential vaccine candidate that could be repurposed for *M. abscessus*.

Novel vaccine candidates with the potential of incorporating VFs also include nucleic acid-based vaccines, such as DNA vaccines. These comprise plasmids encoding antigen/s of interest and employ host cells to synthesize proteins which are recognized by the immune system. Nucleic acid vaccines are advantageous due to the relative ease of design, production and scalability. However, no DNA vaccines have progressed beyond preclinical study within the *M. tb* field—this may be because of their poorly immunogenic nature, or the more complex requirements for vaccine delivery to enter the nucleus and prevent plasmid degradation (Lee et al., 2018; Sefidi-Heris et al., 2020). Recently however, the use of tetrafunctional block copolymers to enhance cell uptake of exogenous nucleic acid has improved our ability to effectively deliver DNA vaccines (Richard-Fiardo et al., 2015). Both preclinical vaccines for *M. abscessus* are DNA formulated with tetrafunctional block polymers, one targeting *M. abscessus* VF PlcC and another targeting MgtC (McIlroy et al., 2009; Le Moigne et al., 2015, 2016). DNA vaccination against *M. abscessus* PlcC appears to provide marginally superior protection to protein-based vaccination, although the immunological mechanisms driving this trend are unclear (Le Moigne et al., 2015). However, both *PlcC* and *MgtC* DNA vaccination have a limited ability to consistently reduce bacterial burden by an appreciable level in both ΔF508 and WT mice across the course of *M. abscessus* infection (Le Moigne et al., 2015, 2016). Recent successes in mRNA vaccination for the Coronavirus Disease 2019 (COVID-19) has also promoted interest in mRNA as a vaccination strategy. This is due to the ease of manufacturing ability and the ability to induce activation of immune cells by multiple cellular pathways in response to mRNA (Brisse et al., 2020). As this area of vaccine design is relatively new, it is likely that continued research will be required to improve the potential of this formulation before use against *M. abscessus*.

Subunit vaccines comprise antigenic proteins from the target species, delivered either through viral vector or with an adjuvant to promote induction of an immune response. The selection of appropriate antigen is a critical consideration for subunit vaccines and may determine the protective outcome. Highly expressed VFs are ideal vaccine candidates, and have been targets for multiple TB vaccines in development (Chakaya et al., 2021). Importantly, antigenic expression for *M. tb* varies over the course of chronic infection and subunit vaccines targeting antigens only expressed at specific stages of infection may lack efficacy (Shi et al., 2004; Rogerson et al., 2006). CF pathogens, such as *P. aeruginosa*, also show marked change in physiology following adaptation to the CF lung, such as development of a mucoid phenotype, reduced VF expression, and loss of motility, suggesting this is a possibility for *M. abscessus* (Winstanley et al., 2016). This obstacle may be overcome through the incorporation of “early” and “late” stage antigens to target a pathogen’s full antigenic repertoire. One such example is CysVac2, a subunit vaccine which combines the secreted mycolyltransferase Ag85B with CysD, a component of the sulfate assimilation pathway expressed highly during chronic infection (Pinto et al., 2013; Counoupas et al., 2016). This highlights the importance of understanding *M. abscessus* antigen expression during infection, which may inform vaccine design.

A particular advantage of subunit vaccines is that the quality and type of the immune response may be more easily modulated through the selection of an adjuvant. Adjuvants, such as these, are most effectively used when their selection is tailored to the variety of immune response generated, which is often independent of the co-administered antigen (Knudsen et al., 2016). A diverse range of adjuvants are currently used in clinical TB vaccine candidates, including TLR agonists and liposomal formulations, such as IC31 and GLA-SE (Stewart et al., 2019; Enriquez et al., 2021). Most of the adjuvants being used in clinical trials for TB encourage the generation of protective T_H_1 and/or T_H_17 immune responses (Stewart et al., 2019). Given this is thought to be an immune correlate of protection against *M. abscessus*, these adjuvants may be useful components of an *M. abscessus* vaccine. Appropriate selection of adjuvant (such as one with a minimal inflammatory profile) may also facilitate non-parenteral vaccine administration, such as vaccine delivery directly to the respiratory mucosa (Ferrell et al., 2021). There is renewed interest in pulmonary vaccination for TB, whereby delivery of the vaccine directly to the lung induces tissue-resident populations capable of rapid response to *M. tb* (Counoupas et al., 2020). Similarly, intranasal vaccination with BCG provides superior protection against *M. tb* infection to subcutaneous vaccination (Perdomo et al., 2016). As our knowledge of immune responses correlated with *M. abscessus* protection increases, this may be a valuable avenue of investigation.

### Reverse Vaccinology for the Development of an *Mycobacterium abscessus* Vaccine

As the field of vaccinology has advanced, so too has the use of bioinformatics approaches to aid vaccination efforts. Increases in genomic sequencing of pathogenic species has given rise to the field of “reverse vaccinology” whereby potential VFs and antigenic targets are discovered through *in silico* analysis of a pathogen’s genome and predicted proteome (Rappuoli, 2000). This method has been relatively underutilized for *M. tb*, likely due to the wealth of *in vitro* knowledge that has emerged from decades of research. A pathogen, such as *M. abscessus*, is an excellent candidate for reverse vaccinology approaches to streamline the identification of potential vaccine candidates, given the continual and growing need for a vaccine against this pathogen. There are numerous studies using pangenome analysis to identify shared VFs across clinical isolates of *M. abscessus*, and this is an ideal starting point for a reverse vaccinology approach (Choo et al., 2014; Davidson et al., 2014; Bryant et al., 2021; Lewin et al., 2021). There is also an extensive range of protein pathway mapping, MHC-II binding prediction, and epitope mapping software currently available which provides greater insight into those proteins likely to yield immunogenic vaccine candidates (Heinson et al., 2015). The reverse vaccinology approach has most recently been used to identify potential VFs and therapeutic targets of *M. abscessus* by development of a novel hierarchical approach; the development of such workflows is beneficial because they may be applied to other pathogens in future (Dar et al., 2021). There is also enormous advantage in a synergistic approach using wet-lab based and *in silico* techniques as a starting point for vaccine candidate identification, as this is based upon the confirmed proteome or secretome of different species. This was recently used by Steindor et al. to identify immunogenic proteins across different clinical isolates of *M. abscessus* and yielded many potential VFs that could be suitable vaccine targets (Steindor et al., 2019). However, current studies on *M. abscessus* reverse vaccinology lack experimental verification of protein immunogenicity; this will be a critical area of future research to determine the best candidates for progression. As we continue to build on our knowledge of this emerging pathogen, reverse vaccinology will become an essential tool in antigenic discovery for the development of subunit vaccines.

## Conclusion

In recent years, we have elevated our understanding of *M. abscessus* from a relatively innocuous environmental organism to a formidable evolving true pathogen with a range of immune modulatory mechanisms to facilitate its survival within the host. These include VFs with homology to well-known mycobacterial VFs in pathogenic species, such as *M. tb*, in addition to non-mycobacterial VFs which bear striking resemblance to those present within other CF species, such as *B. cenocepacea* and *P. aeruginosa*. These VFs have a range of immune modulatory mechanisms to enhance *M. abscessus* survival within the host: promoting phagosomal escape, restriction of phagosomal acidification, enhancement of bacterial cording, and immune masking to escape detection. These, in conjunction with the extensive antibiotic resistance of this pathogen, have likely contributed to the increasing global incidence and severity of pulmonary *M. abscessus*. However, through increased understanding of *M. abscessus* virulence, we have uncovered potential vaccine candidates and novel drug targets, bringing us closer to novel prevention and eradication strategies for this pathogen. Development of a vaccine may also be guided effectively by lessons learned from decades of research into other pathogenic Mycobacteria, such as *M. tb*. Continued research into the virulence and immune correlates of protection in *M. abscessus* pathogenesis using biologically relevant animal models will provide insight into the best strategies to adopt for the development of an *M. abscessus* vaccine, to improve the quality of life of susceptible populations.

## Author Contributions

KF and CC conceptualized the idea of the review article. KF wrote the first draft of the manuscript. KF, MJ, JT, and CC provided revision to the scientific content of the final manuscript. All authors contributed to the article and approved the submitted version.

## Funding

JT is supported by the NHMRC Centre of Research Excellence in Tuberculosis Control (1153493).

## Conflict of Interest

The authors declare that the research was conducted in the absence of any commercial or financial relationships that could be construed as a potential conflict of interest.

## Publisher’s Note

All claims expressed in this article are solely those of the authors and do not necessarily represent those of their affiliated organizations, or those of the publisher, the editors and the reviewers. Any product that may be evaluated in this article, or claim that may be made by its manufacturer, is not guaranteed or endorsed by the publisher.
